# MEK Inhibitors, Novel Anti-Adhesive Molecules, Reduce Sickle Red Blood Cell Adhesion *In Vitro* and *In Vivo,* and Vasoocclusion *In Vivo*

**DOI:** 10.1371/journal.pone.0110306

**Published:** 2014-10-20

**Authors:** Rahima Zennadi

**Affiliations:** Division of Hematology and Duke Comprehensive Sickle Cell Center, Department of Medicine, Duke University Medical Center, Durham, North Carolina, United States of America; University of Missouri, United States of America

## Abstract

In sickle cell disease, sickle erythrocyte (SSRBC) interacts with endothelial cells, leukocytes, and platelets, and activates coagulation and inflammation, promoting vessel obstruction, which leads to serious life-threatening complications, including acute painful crises and irreversible damage to multiple organs. The mitogen-activated protein kinase, ERK1/2, is abnormally activated in SSRBCs. However, the therapeutic potential of SSRBC ERK1/2 inactivation has never been investigated. I tested four different inhibitors of MEK1/2 (MEK), the kinase that activates ERK1/2, in a model of human SSRBC adhesion to TNFα-activated endothelial cells (ECs). SSRBC MEK inhibition abrogated adhesion to non-activated and TNFα-activated ECs to levels below baseline SSRBC adhesion to non-activated ECs *in vitro*. SSRBC MEK inhibition also prevented SSRBCs from activating naïve neutrophils to adhere to endothelium. To determine the effect of MEK inhibitors on SSRBC adherence *in vivo*, sham-treated or MEK inhibitor-treated SSRBCs were infused to nude mice previously treated with TNFα. Sham-treated SSRBCs displayed marked adhesion and occlusion of enflamed vessels, both small and large. However, SSRBC treatment with MEK inhibitors *ex vivo* showed poor SSRBC adhesion to enflamed vessels with no visible vasoocclusion *in vivo*. In addition, MEK inhibitor treatment of SSRBCs reduced SSRBC organ trapping and increased the number of SSRBCs circulating in bloodstream. Thus, these data suggest that SSRBC ERK1/2 plays potentially a critical role in sickle pathogenesis, and that MEK inhibitors may represent a valuable intervention for acute sickle cell crises.

## Introduction

In sickle cell disease (SCD), sickle red blood cells (SSRBCs) have been postulated to play a central role in vasoocclusion by adhering to the endothelium, leukocytes and platelets in capillaries and small vessels, and activating coagulation and inflammation. Vasoocclusion impairs blood flow leading to acute painful vasoocclusive crises “pain crises” and ischemic organ and tissue damage [1]–[6].

To date, investigators have targeted SSRBCs and explored multiple approaches to treating acute vasoocclusive crises. Therapies that focus on ameliorating SSRBC dehydration [7]–[11], interfering with chemical-physical processes during erythrocyte-endothelial adhesion events [12], or targeting RBC adhesion molecules [13]–[15] to prevent RBC-endothelial cell (EC) interactions have shown little to no therapeutic benefit. Nevertheless, despite the known contribution of SSRBC adhesiveness to vasoocclusion, we lack treatments that specifically target intracellular signaling mechanisms critical for SSRBC adhesion [1], [14], [16]–[18]. An in-depth understanding of signaling pathways in SSRBCs that mediate adhesion at both biochemical and physiological levels will be required to successfully exploit these pathways for therapeutic purposes and to develop efficacious pathway-selective drugs with minimal side effects.

We have recently discovered that the mitogen-activated protein kinase (MAPK), ERK1/2, is pathophysiologically activated in SSRBCs, but not in normal RBCs [16]–[18]. Patients with SCD manifest chronic and acute activation of inflammation, as evidenced by a statistically significant and distinct rise in plasma levels of several pro-inflammatory cytokines such as tumor necrosis factor alpha (TNFα), interleukin 1beta (IL-1β), IL-4, IL-6, IL-8 and interferon gamma (IFNγ) either at steady state or during painful crisis [19]–[21]. Because increased proinflammatory cytokines can up-regulate endothelial expression of ligands for red blood cell adhesion molecules, it remains unknown whether inhibition of ERK1/2 activation in SSRBCs has therapeutic benefits in reducing SSRBC adhesion and vasoocclusion in the presence of inflammation *in vivo*. Therefore, I investigated the therapeutic potential of SSRBC ERK1/2 inactivation using inhibitors of MEK1/2 (MEK), the kinase that activates ERK1/2, to inhibit both human SSRBC adhesion to TNFα-activated ECs and SSRBC’s ability to activate neutrophil adhesion *in vitro,* and reduce vasoocclusion *in vivo*. These studies were undertaken because MEK inhibitors have been developed for human use [22]–[25], and the MEK inhibitor, trametinib (GSK1120212), has been recently approved by the U.S. Food and Drug Administration (FDA) for treatment of melanoma [24], [25]. Thus, my studies may lead to rapid development of existing MEK inhibitors as a novel therapeutic approach for acute sickle cell crises.

## Materials and Methods

### Endothelial cells

Human umbilical vein endothelial cells (HUVECs, ATCC), the murine endothelial cell line EOMA (ATCC, Manassas, VA), which exhibits properties characteristic of microvascular endothelial cells, and human dermal microvascular endothelial cells (HMVECs-d) (Lonza, Walkersville, MD) were grown as monolayers in EBM2 medium (Clonetics, Walkersville, MD) supplemented with EGM2 (Clonetics) [14], [17]. For *in vitro* flow chamber adhesion experiments, ECs were cultured until they reached confluence on clear glass slides pre-coated with 2% gelatin.

### Collection, preparation and treatment of RBCs

Blood samples obtained from human participants has been approved by Duke University's Institutional Review Board (IRB), and written informed consent has been obtained from the participants. Blood samples were obtained from adult SCD patients, 52% of whom were male, and from adult healthy donors. SCD patients were of age between 21 and 69 years old with a mean age of 38.5±3 years old. All SCD patients had not been transfused for at least three months and had not experienced acute vasoocclusive crises for three weeks, and half of these patients were on hydroxyurea. Blood samples were collected and packed RBCs were separated as previously described in detail [17]. Packed RBCs were treated with various reagents to affect protein phosphorylation. RBCs were treated at 37°C for 1 h with one of the following reagents: 100 nM of the MEK inhibitor U0126 (Calbiochem, La Jolla, CA); 100 nM of the MEK inhibitor RDEA119 (CGeneTech, Inc., Indianapolis, IN); 100 nM of the MEK inhibitor trametinib (GSK1120212) (Active Biochemicals Co., Wanchai, Hong Kong); 100 nM of the MEK inhibitor AZD6244 (Selleckchem, Houston, TX); and 10 µM of the tyrosine kinase inhibitor damnacanthal (Enzo Life Sciences International, Inc., Plymouth Meeting, PA). Sham-treated RBCs were incubated with the same buffer and vehicle but without the active agent. Treated RBCs were washed 5 times with 4 ml PBS with Ca^2+^ and Mg^2+^. In some *in vitro* and *in vivo* adhesion studies, RBCs were fluorescently labeled as described previously in detail [14], [17], [26].

### Neutrophil separation and activation of adhesion by SSRBCs

Separation of peripheral blood mononuclear cells (PBMCs) from neutrophils and red cells from blood from healthy donors was performed as previously described in detail [16]. Pellets composed of neutrophils and RBCs were very gently washed once with PBS to avoid activation of neutrophils. Cells were gently re-suspended in PBS and mixed with an equal volume of 3% dextran. Tubes were set upright for 20 minutes at room temperature. Neutrophil-rich upper layer was collected, RBCs contaminating neutrophils were lysed with RBC lysis buffer, and neutrophils were then washed and fluorescently labeled. In parallel, packed RBCs were sham-treated or treated with a MEK inhibitor, then extensively washed prior to co-incubation for 30 min with fluorescence-labeled PMNs and performance of PMN adhesion assays as described previously [14], [16].

In some experiments, HUVECs treated or not with 10 ng/ml recombinant human TNFα (Sigma-Aldrich, St. Louis, MO) for 4 h, were co-incubated for 30 min at 37°C with washed sham-treated or U0126-treated RBCs. HUVECs were then washed extensively to remove non-adherent RBCs prior to PMN adhesion assays.

### In vitro flow chamber adhesion assays

Adherent HUVECs, HMVECs-d or EOMA cells were non-treated or treated with 10 ng/ml recombinant human TNFα for 4 h at 37°C. ECs were then washed three times with 20 ml PBS prior to adhesion assays. RBC or PMN adhesion to washed non-treated or TNFα-treated ECs was assayed in graduated height flow chambers as described previously in detail [14].

### Mice

Animal work was approved by the Institutional Animal Care and Use Committee (IACUC) at Duke University. All animal experiments were carried out in strict accordance with and following the National Institutes of Health (NIH) guidelines and recommendations for the Care and Use of Laboratory Animals. The protocol was approved by the Committee on the Ethics of Animal Experiments of Duke University (Permit Number: A023-12-01). All surgery was performed under anesthesia by intra-peritoneal injection of 100 mg/kg of ketamine (Abbott Laboratory, Chicago, IL) and 10 mg/kg of xylazine (Bayer, Shawnee Mission, KS), and all efforts were made to minimize suffering. Female athymic homozygous nude (nu−/nu−) mice, 8–12 weeks of age, were bred at Duke University and housed at the vivarium at Duke University.

### Window chamber surgery, RBC infusion and intravital microscopy

Dorsal skin-fold window chamber surgery was performed on anesthetized nude mice as described previously [1], [17], [27]–[29]. Animals were used three days following surgery. To determine the therapeutic potential of SSRBC ERK1/2 inactivation *in vivo*, nude mice with dorsal skin-fold window chambers were first injected intraperitoneal with 500 ng murine recombinant TNFα (Peprotech, Rocky Hill, NJ), to induce inflammation. Four hours later, animals were anesthetized as previously described [17], then infused with sham-treated or MEK inhibitor-treated Dil-labeled human SSRBCs (300 µl, hematocrit [Hct] 50% in saline). All infusions were through the dorsal tail vein. Intravital microscopy through the dorsal skin-fold implants was conducted as described before in detail [17]. SSRBC adhesion and blood flow dynamics were observed in subdermal vessels using 10× and 20× magnifications, and at least 180 venule segments were videotaped for a period of at least 60 minutes.

Human SSRBC adhesion was quantified on still images by measuring the fluorescence intensity (pixels) of adherent fluorescently labeled SSRBCs using ImageJ software downloaded from the NIH website. The values abtained from the vessels analyzed in the accompanying videos were averaged among groups of animals (n = 5) to obtain mean fluorescence intensity and for statistical analysis. The data are presented as the fluorescence intensity of adherent SSRBCs (pixels). The percentage of vessels occupied by adherent SSRBCs was calculated by dividing the number of vessels (all vessels, small and large) supporting adherent SSRBCs by the total number of vessels recorded, then averaged among groups of animals (n = 5). The percentages of vessels with normal blood flow, slow blood flow and no blood flow were also calculated by dividing of number of vessels with normal blood flow, slow blood flow, and no blood flow (occluded vessels) by the total number of vessels recorded, respectively.

### Histology

Animals were sacrificed 2 hours post-injection of fluorescence-labeled SSRBCs, and organs were collected and snap frozen in OCT media. Tissue section preparation was performed as previously described in detail [17]. Three random fields were imaged for each section of each organ, and fluorescence intensity for each field was quantified using ImageJ software. The values were averaged for the three fields to obtain mean fluorescence intensity. The mean fluorescence values were averaged among groups of animals (n = 3) for statistical analysis.

### Flow cytometric analysis

Fluorescence-labeled sham-treated or MEK inhibitor-treated SSRBCs from a single donor were infused to TNFα-treated nude mice. Blood samples were collected 1, 10 and 20 min post-injection of SSRBCs and analyzed for the presence of human SSRBCs using a FACScan flow cytometer (Becton Dickinson, San Jose, CA) [17].

### Statistical analysis

Results using sham and treated RBCs were compared only with results using the same donor sample, because SSRBC adhesion varies greatly among SCD patients [14]. Data were compared using parametric analyses (GraphPad Prism 5 Software, San Diego, CA), including repeated and non-repeated measures of analysis of variance (ANOVA). One-way and two-way ANOVA analyses were followed by Bonferroni corrections for multiple comparisons (multiplying the *p* value by the number of comparisons). A *p* value <0.05 was considered significant.

## Results

### MEK inhibition inhibits SSRBC adhesion to endothelial cells in vitro

**Figure 1:**
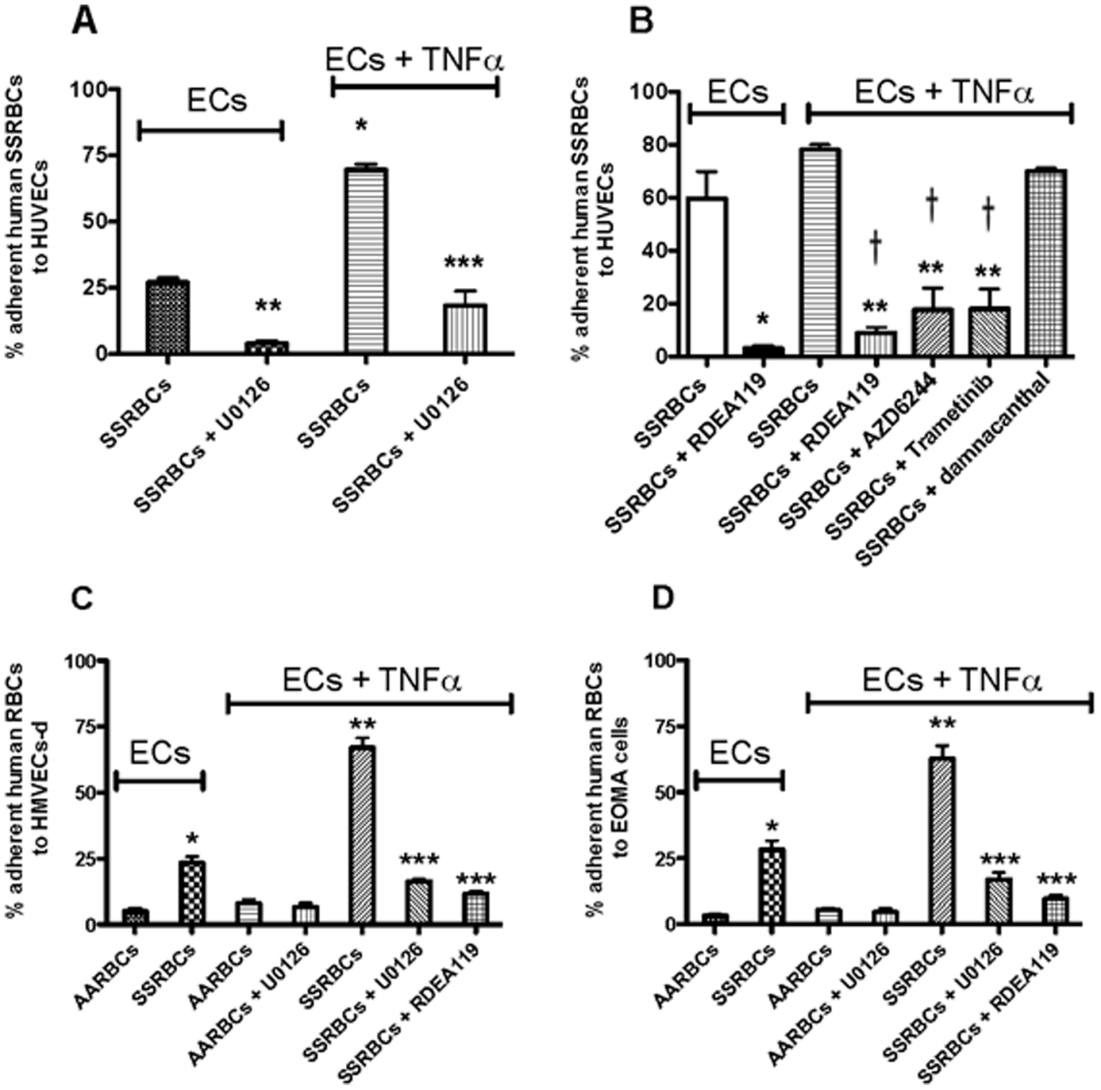
MEK inhibition down-regulates SSRBC adhesion to both non-activated and activated endothelial cells *in vitro*. The effects of MEK inhibitors on SSRBC adhesion to HUVECs, HMVECs-d and EOMA cells was tested in intermittent flow condition assays at different shear stresses *in vitro*. Results are presented as % adherent SSRBCs at a shear stress of 2 dynes/cm^2^. **A.** SSRBCs were sham-treated or treated with 100 nM MEK inhibitor U0126 prior to adhesion assays to non-treated and TNFα-treated HUVECs. *: *p*<0.0001 compared to sham-treated SSRBCs adherent to non-treated HUVECs; **: *p*<0.001 compared to sham-treated SSRBCs adherent to non-treated HUVECs; ***: *p*<0.0001 compared to sham-treated SSRBCs adherent to TNFα-treated HUVECs. Error bars show standard error mean (SEM) of 4 different experiments. **B.** SSRBCs were sham-treated, or treated with 100 nM RDEA119, 100 nM AZD6244, 100 nM trametinib, or 10 µM damnacanthal prior to adhesion assays to non-treated and TNFα-treated HUVECs. *: *p*<0.0001 compared to sham-treated SSRBCs adherent to non-treated HUVECs; **: *p*<0.0001 compared to sham-treated SSRBCs adherent to TNFα-treated HUVECs; and ^†^: *p*<0.001 compared to sham-treated SSRBCs adherent to non-treated HUVECs. Error bars show SEM of 3 different experiments. **C**–**D.** SSRBCs and normal RBCs (AARBCs) were sham-treated, or treated with 100 nM U0126 or 100 nM RDEA119 prior to adhesion assays to non-treated and TNFα-treated HMVECs-d (**C**) and EOMA cells (**D**). *: *p*<0.0001 compared to sham-treated AARBCs adherent to non-treated HMVECs-d (**C**) and EOMA cells (**D**); **: *p*<0.0001 compared to sham-treated SSRBCs adherent to non-treated HMVECs-d (**C**) and EOMA cells (**D**); and ***: *p*<0.001 compared to sham-treated SSRBCs adherent to TNFα-treated HMVECs-d (**C**) and EOMA cells (**D**). Error bars show SEM of 3 different experiments for **C** and **D**.

We have previously shown that ERK1/2 is abnormally activated in SSRBCs, but not in normal RBCs [18]. Because inflammation can initiate vasoocclusion [30]–[33], I first determined the relevance of SSRBC ERK1/2 inactivation in adhesion to TNFα-activated HUVECs *in vitro.* To inhibit ERK1/2, I used the MEK (kinase that directly activates ERK1/2) inhibitor, U0126, developed for animal use, and the MEK inhibitors, RDEA119, trametinib and AZD6244 developed for human use. The degree of adhesion of sham-treated SSRBCs to non-activated HUVECs varied from patient to patient (n = 7) and such variability in adhesion was unrelated to whether the patient was on hydroxyurea or not [14]. SSRBC adhesion was moderate (27±1.7%; n = 4; Figure 1A) to marked (60±10.1%; n = 3; Figure 1B) under intermittent flow conditions at a shear stress of 2 dynes/cm^2^. Adhesion of sham-treated SSRBCs to TNFα-activated HUVECs increased by 2.6±0.17-fold (*p*<0.05) and 1.4±0.23-fold (*p*>0.05) over baseline adhesion as shown in Figures 1A and 1B, respectively. However, treatment of SSRBCs with 100 nM U0126 or RDEA119 significantly inhibited adhesion of SSRBCs to non-activated HUVECs by 86±4.5% (*p*<0.001) and 95±1.2% (*p*<0.0001), respectively (Figures 1A and 1B). Similarly, 100 nM U0126, RDEA119, AZD6244 or trametinib also decreased SSRBC adhesion to TNFα-activated HUVECs to levels below baseline adhesion of sham-treated SSRBCs to non-activated HUVECs (*p*<0.0001 for each of the MEK inhibitors) (Figures 1A and 1B). In contrast, 10 µM damnacanthal, a highly potent and selective inhibitor of tyrosine kinase p56^Lck^ [34], failed to inhibit SSRBC adhesion to TNFα-activated HUVECs (Figure 1B).

Because vascular bed is known to be involved in vasoocclusive pain crises, and prior to conduct our *in vivo* studies using nude mouse dorsal skin microvasculature, I next tested the effect of MEK inhibition on adhesion of human SSRBCs to human and mouse microvascular ECs, HMVECs-d and EOMA cells, respectively, non-treated or treated with TNFα, then washed. Less than 25% of non-treated SSRBCs adhered to non-activated HMVECs-d at a shear stress of 2 dynes/cm^2^ (Figure 1C), and adhesion of these sickle cells increased significantly when HMVECs-d were treated with TNFα (*p*<0.0001). Treatment of SSRBCs with U0126 or RDEA119 again significantly inhibited adhesion of SSRBCs to TNFα-activated HMVECs-d to levels below baseline adhesion of SSRBCs to non-activated HMVECs-d (*p*<0.0001 for each inhibitor; Figure 1C). However, basal normal RBCs (AARBCs) adherence to non-activated HMVECs-d was much lower than baseline adhesion of SSRBCs (*p*<0.0001), and treatment of HMVECs-d with TNFα did not support significant increased adhesion of AARBCs (*p*>0.05 compared to AARBC adhesion to non-activated HMVECs-d). In addition, although AARBCs adhered to some degree to TNFα-activated HMVECs-d, U0126 had no effect on such adhesion (Figure 1C).

Similar data were obtained when EOMA cells were used. SSRBCs adhered to some degree to non-activated EOMA cells at a shear stress of 2 dynes/cm^2^ (Figure 1D). However, SSRBC adhesion to TNFα-activated EOMA cells increased by 2.2±0.09-fold over baseline SSRBC adhesion to non-activated EOMA cells (*p*<0.0001). U0126 or RDEA119 once more inhibited SSRBC adhesion to TNFα-activated EOMA cells to levels below baseline adhesion of sham-treated SSRBCs to non-activated EOMA cells (*p*<0.0001 for each inhibitor). In contrast, AARBCs adhered weakly to non-activated EOMA cells compared to basal adhesion of SSRBCs, and adhesion of AARBCs to TNFα-activated EOMA cells was slightly, but not significantly, up-regulated. The MEK inhibitor U0126 did not affect adhesion of these normal red cells to TNFα-activated EOMA cells (Figure 1D). Together, these data strongly suggest that MEK-dependent ERK1/2 inactivation in SSRBCs completely inhibits SSRBC adhesion to both non-activated and activated microvascular endothelial cells, an effect that does not require increased activation of SSRBC ERK1/2.

### MEK inhibition prevents SSRBCs from stimulating neutrophil adhesion in vitro

**Figure 2:**
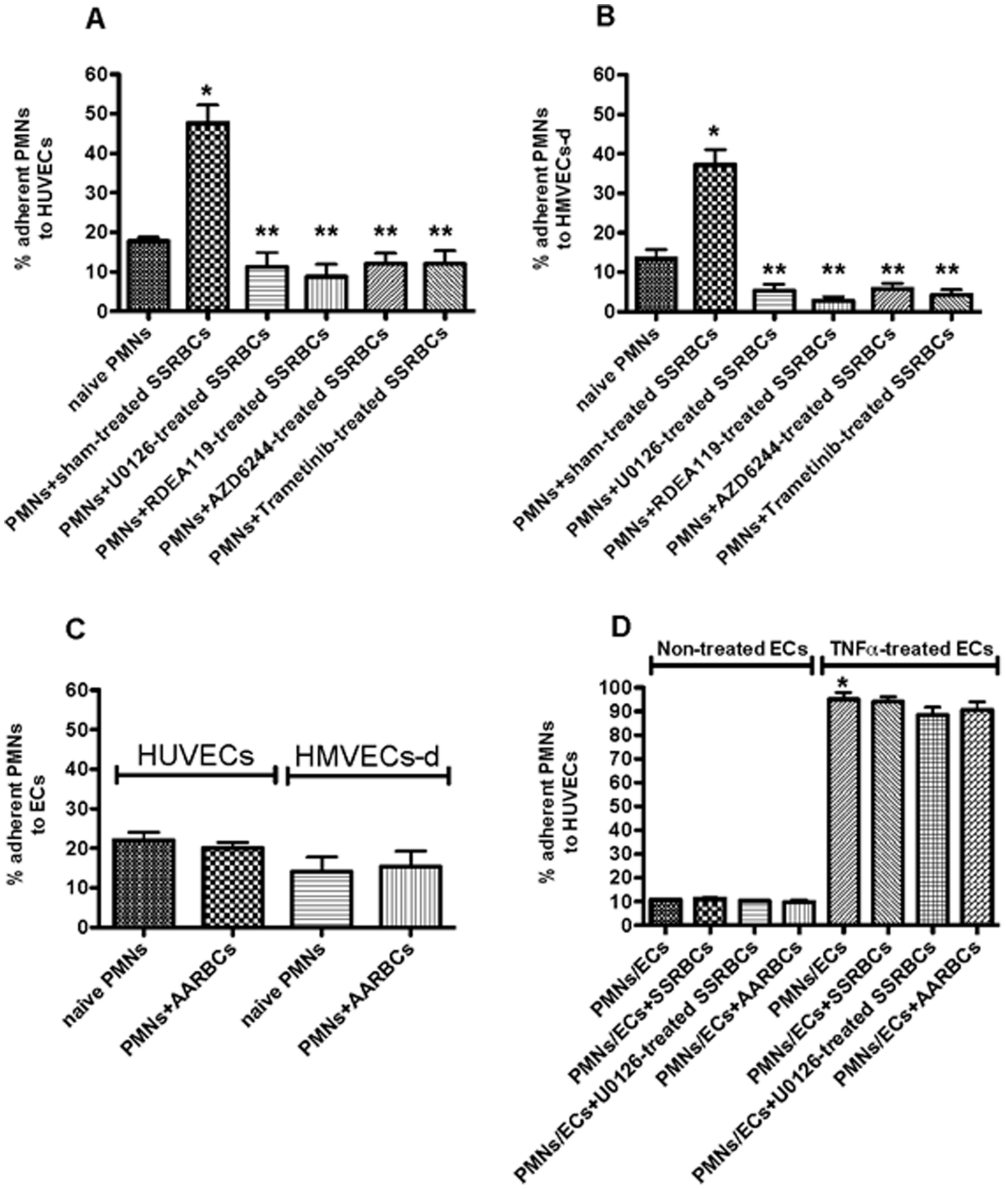
MEK inhibition prevents SSRBCs from activating neutrophil adhesion. The effect of MEK inhibitors on the ability of SSRBCs to stimulate neutrophil (PMN) adhesion to ECs was tested. **A** and **B**. SSRBCs (n = 8) were sham-treated or treated with 100 nM MEK inhibitor U0126, RDEA119, AZD6244 or trametinib. Washed treated SSRBCs were then co-incubated with ABO-matched naïve PMNs isolated from healthy donors (n = 8), prior to testing adhesion of PMNs to HUVECs (**A;** n = 4) and HMVECs-d (**B;** n = 4) in intermittent flow condition assays at different shear stresses. **C.** AARBCs (n = 3) were sham-treated, washed, and then co-incubated with ABO-matched naïve PMNs isolated from healthy donors (n = 3), prior to testing adhesion of PMNs to HUVECs and HMVECs-d at different shear stresses. **D.** Non-treated and TNFα-treated HUVECs were co-incubated with sham-treated SSRBCs, U0126-treated SSRBCs or sham-treated AARBCs. HUVECs were then washed free of non-adherent RBCs, and tested for their ability to support adhesion of PMNs (n = 3). Results are presented as % adherent PMNs at a shear stress of 1 dyne/cm^2^. *:*p*<0.0001 compared to adhesion of naïve PMNs (PMNs only) to non-treated ECs; and **:*p*<0.0001 compared to adhesion of PMNs stimulated with SSRBCs (PMNs+SSRBCs). Error bars show SEM of 4 different experiments for **A and B**, and 3 different experiments for **C** and **D**.

Patients with SCD have high counts of activated polymorphonuclear neutrophils (PMNs) [35], and leukocytosis, in the absence of infection, correlates well with both clinical severity and vasoocclusion [35], [36]. I examined whether SSRBCs activate PMNs to adhere to ECs and whether MEK inhibitors prevent such effect. Washed sham-treated or MEK-inhibitor treated SSRBCs were co-incubated with isolated naïve PMNs prior to PMN adhesion assays. Naïve PMNs adhered to some degree to both HUVECs and HMVECs-d (18±1% and 14±2.2% adherent PMNs, respectively) at a shear stress of 1 dyne/cm^2^ (Figures 2A and 2B). PMN adhesion to HUVECs (n = 4) and HMVECs-d (n = 4) following PMN co-incubation with sham-treated SSRBCs, increased by a 2.7±0.2-fold (*p*<0.0001) and 3±0.5-fold (*p*<0.0001), respectively, over baseline adhesion of naïve PMNs. However, SSRBC MEK inactivation with U0126, RDEA119, AZD6244 or trametinib inhibited the ability of SSRBCs to activate naïve PMN adhesion to either HUVECs or HMVECs-d, and PMN adhesion decreased to levels below baseline adhesion of native PMNs (*p*<0.0001 for each MEK inhibitor) (Figures 2A and 2B). In contrast, AARBCs were not able to up-regulate PMN adhesion to HUVECs and HMVECs-d compared to adhesion of naïve PMNs (Figure 2C). These data suggest that SSRBC MEK-dependent ERK1/2 inactivation completely prevents SSRBCs from stimulating PMN adhesion to microvascular endothelial cells.

Furthermore, to evaluate whether SSRBCs can also induce endothelial changes to support increased PMN adhesion, HUVECs were co-incubated with SSRBCs or AARBCs. After washing to eliminate non-adherent SSRBCs or AARBCs, adhesion to HUVECs of naïve PMNs not previously co-incubated with RBCs did not significantly increased compared to naïve PMN adhesion to HUVECs not co-incubated with RBCs (*p*>0.05, Figure 2D). Naïve PMNs also adhered similarly to HUVECs co-incubated with U0126-treated SSRBCs and HUVECs not co-incubated with RBCs (*p*>0.05, Figure 2D). We also evaluated whether SSRBC co-incubation with TNFα-treated HUVECs can affect adhesion of naïve PMNs to HUVECs. Naïve PMNs adhered strongly to TNFα-activated HUVECs compared to adhesion of PMNs to non-activated HUVECs (*p*<0.0001, Figure 2D). After washing to eliminate non-adherent SSRBCs or AARBCs, no significant change in adhesion of naïve PMNs to TNFα-activated HUVECs was observed (*p*>0.05 compared to PMN adhesion to TNFα-activated HUVECs not co-incubated with RBCs, Figure 2D). These data suggest that interactions of SSRBCs with endothelial cells failed to induce changes in endothelial cells to support increased PMN adhesion.

### MEK inhibition reduces SSRBC adhesion to the vascular endothelium and vasoocclusion in vivo

These experiments were designed specifically to study the effect of MEK inhibitors on human SSRBC adhesion and vasoocclusion *in vivo* in the absence of the potential effect of these inhibitors on non-human RBCs, including murine RBCs, endothelium, leukocytes or platelets, and to determine whether SSRBC MEK-dependent ERK1/2 inactivation has therapeutic potential in reducing RBC adhesion *in vivo*. Normal and SS human RBC preparations contained 0.25±0.8×10^6^/µl RBCs and 0.1±0.01×10^6^/µl RBCs, respectively, and both showed unmeasurable (0 cells/µl) leukocytes or platelets, making it unlikely that human leukocytes and platelets could participate in SSRBC adhesion and vasoocclusion in my model. In addition, in these studies, the quantity of human RBCs infused to animals never exceed 10% of the total circulating RBCs, assuming that the mouse blood volume is 1.5 ml, thereby minimizing any possible rheological effects attributable to increased hematocrit [37]. RBCs in these small concentrations should also not influence vascular regulatory mechanisms or O_2_ delivery.

**Figure 3:**
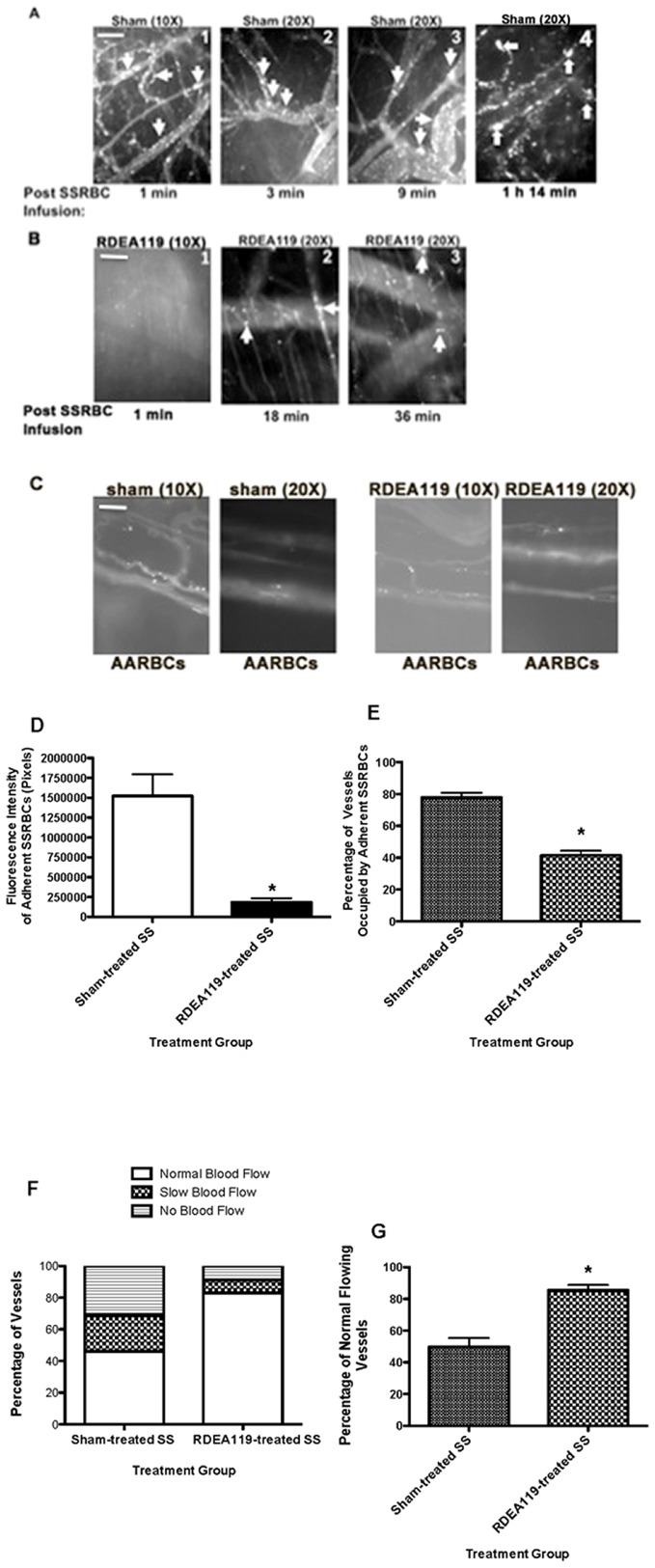
The MEK inhibitor RDEA119 reduces SSRBC adhesion in enflamed vessels and vasoocclusion *in vivo*. Nude mice implanted with dorsal skin-fold window chambers were injected with murine TNFα. Four hours later, intravital microscopic observations of post-capillary venules and arterioles using 10× and 20× magnifications were conducted through the window chamber immediately after infusion of fluorescently labeled sham- (**A**; panels 1, 2, 3 and 4) or RDEA119-treated (**B**; panels 1, 2 and 3) human SSRBCs (n = 5), or sham-treated (**C**; two panels on the left) or RDEA119-treated (**C**; 2 panels on the right) human normal (AA) RBCs (n = 3). Vessels without adherent cells appear gray, due to the rapidly moving of fluorescence labeled RBCs. Adhesion of human SSRBCs in enflamed vessels and vasoocclusion are indicated with arrows. Sham-treated human SSRBCs showed marked adhesion with intermittent vasoocclusion (**A**), whereas RDEA119-treated SSRBCs showed little adhesion to enflamed vessel walls (**B**). Sham-treated and RDEA119-treated human AARBCs showed no adhesion to venule walls (**C**). Scale bar = 50 µm. **D.** Video frames showing vessel and arteriole segments were used to quantify adhesion in venules and arterioles of animals occupied by SSRBCs (n = 5 for each treatment). Adhesion of fluorescently labeled sham-treated SSRBCs (sham-treated SS) and RDEA119-treated SSRBCs (RDEA119-treated SS) in all vessels and arterioles recorded presented as fluorescence intensity of adherent SSRBCs (pixels). **E**–**G.** The values of at least 180 segments of vessels and arterioles were analyzed and averaged among groups of animals (n = 5) to represent percentage of vessels occupied by adherent SSRBCs (**E**); percentage of vessels with normal blood flow, slow blood flow and no blood flow (**F**); and percentage of normal flowing vessels (**G**). Error bars show SEM of 5 different experiments for each treatment. *: *p*<0.001 compared to sham-treated SSRBCs regardless of the vessel diameter within the ranges specified for **D**, **E** and **G**.

To determine the therapeutic relevance of SSRBC MEK inactivation *in vivo*, human SSRBCs were sham-treated or treated with the MEK inhibitors, RDEA119 or U0126, *ex vivo*, washed, then adoptively transferred to nude mice previously treated with TNFα. Intravital microscopy observation of enflamed venules and arterioles visible through the dorsal skin-fold window chamber for at least 1 hour, showed that sham-treated human SSRBCs adhered to 78±3% of vessels, both small and large, and arterioles with mean diameter = 29±1.57 µm [Figures 3A (panels 1, 2, 3 and 4) and 3D–E; Movie S1]. SSRBC adhesion occurred 1–4 min following SSRBC infusion, and progressively occluded micro-vessels at junctional and non-junctional points, and at curved and straight segments with evident blood stasis (Figures 3A, 3F and 3G). In sharp contrast, RDEA119-treated SSRBCs demonstrated less frequent adhesion, which occurred in vessels much smaller in diameter than vessels supporting sham-treated SSRBC adhesion, with mean diameter no larger than 13±1.32 µm [Figures 3B (panels 1, 2 and 3) and 3D–E; Movie S2]. Adhesion of RDEA119-treated SSRBCs was reduced by 88% compared to sham-treated SSRBCs (n = 5; *p*<0.0001) (Figures 3A, 3B and 3D). As a result of RDEA119 treatment, SSRBCs promoted occasional small vessel obstruction, and normal blood flow was restored in 86±3.3% of vessels and arterioles recorded compared to 50±5.5% of vessels with normal blood flow in animals infused with sham-treated cells (*p*<0.0001; Figures 3A, 3B, 3F and 3G). The involvement of ERK1/2 in normal RBCs adhesion *in vivo* was also tested. Sham-treated and RDEA119-treated normal human RBCs showed no real adherence in enflamed vessels [Figure 3C (panels 1, 2, 3 and 4); Movie S3], further confirming our previous data that ERK1/2 is inactived in normal RBCs [18].

**Figure 4:**
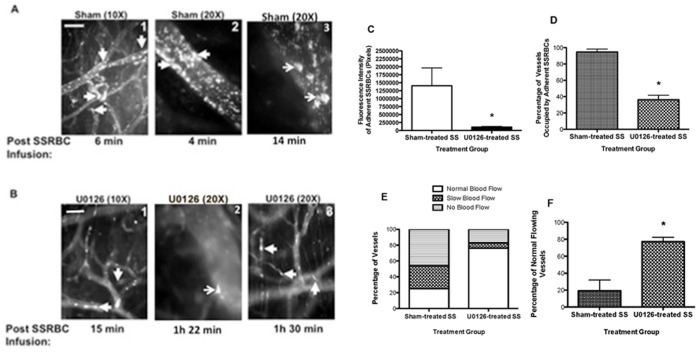
The MEK inhibitor U0126 abrogates SSRBC adhesion and vasoocclusion *in vivo*. Nude mice implanted with dorsal skin-fold window chambers were injected with murine TNFα. Four hours later, intravital microscopic observations of post-capillary venules using 10× and 20× magnifications were conducted through the window chamber immediately after infusion of fluorescently-labeled sham- (**A**; panels 1, 2 and 3) or U0126-treated (**B**; panels 1, 2 and 3) human SSRBCs (n = 5). SSRBC adhesion and vasoocclusion are indicated with arrows. While sham-treated SSRBCs adhered markedly to venule walls promoting vasoocclusion, U0126 treatment of SSRBCs significantly reduced SSRBC adhesion and stasis. Scale bar = 50 µm. **C.** Video frames showing vessel segments were used to quantify adhesion in venules of animals occupied by SSRBCs (n = 5 for each treatment). Adhesion of fluorescently labeled sham-treated SSRBCs (sham-treated SS) and U0126-treated SSRBCs (U0126-treated SS) observed in all vessels recorded presented as fluorescence intensity of adherent SSRBCs (pixels). **D**–**F.** The values of at least 35 segments of vessels were analyzed and averaged among groups of animals (n = 5) to represent percentage of vessels occupied by adherent SSRBCs (**D**); percentage of vessels with normal blood flow, slow blood flow and no blood flow (**E**); and percentage of normal flowing vessels (**F**). Error bars show SEM of 5 different experiments for each treatment condition. *: *p* = 0.0103 (**C**) and *p*<0.0001 (**D** and **F**) compared to sham-treated SS regardless of the vessel diameter within the ranges specified.

Similar anti-SSRBC adhesive benefit was obtained with U0126. Sham-treated SSRBCs showed persistence in SSRBC adhesion in both small and large diameter vessels with mean diameter = 35±9.2 µm promoting vasoocclusion [Figures 4A (panels 1, 2 and 3) and 4C]. However, treatment of SSRBCs with U0126 significantly reduced SSRBC adhesion, which was observed in much smaller vessels with mean diameter = 20±3.2 µm, and less frequent occlusion of only small postcapillary vessels (*p* = 0.0103) compared to sham-treated SSRBCs [Figures 4B (panels 1, 2 and 3) and 4C]. While sham-treated SSRBCs adhered to 94.75±3.5% of total vessels and arterioles recorded, U0126-treated SSRBCs occupied only 36±5.4% of vessels (n = 5; *p*<0.0001; Figures 4A, 4B and 4D). SSRBC MEK inhibition led to improved SSRBC circulatory behavior and blood flow in 77±5.2% of vessels in animals infused with U0126-treated cells as opposed to 19±12.6% of vessels with normal blood flow in mice infused with sham-treated SSRBCs (*p*<0.001) (Figures 4A, 4B, 4E and 4F). Together, these data strongly suggest that SSRBC MEK-dependent ERK1/2 inactivation effectively reduces vasoocclusion and restores normal blood flow in vessels by at least down-regulating SSRBC adhesive function.

### MEK inhibition in SSRBCs reduces human SSRBC organ trapping

**Figure 5:**
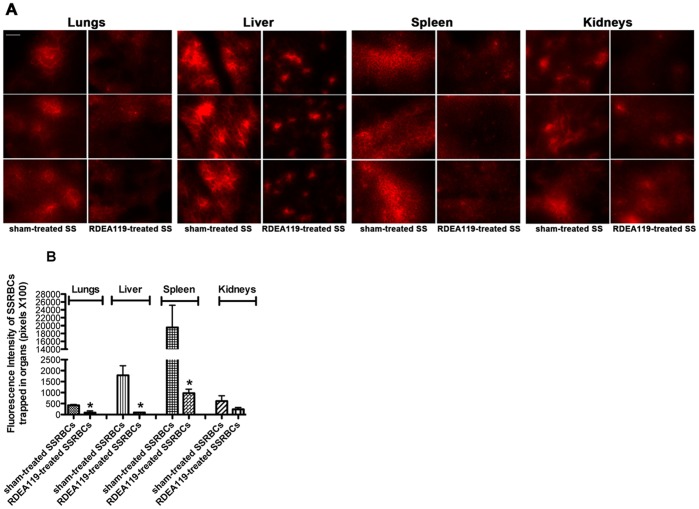
The MEK inhibitor RDEA119 diminishes human SSRBC organ infiltration and trapping. Nude mice preinjected with murine TNFα were infused 4 hours later with sham-treated SSRBCs (sham-treated SS) or RDEA119-treated SSRBCs (RDEA119-treated SS) (50% hematocrit; n = 3 for each treatment). **A and B**. Two hours following RBC infusion, animals were sacrificed and the lungs, liver, spleen and kidneys were harvested. Tissue sections were analyzed and quantitated for the presence of fluorescently labeled SSRBCs. The three panels for each treatment in **A** represent three different experiments with similar results. Scale bar = 150 µm. **B.** The effect of RDEA119 treatment on SSRBC trapping in organs was quantitated and presented as fluorescence intensity of fluorescence-labeled SSRBC trapped in organs (pixels). RDEA119 treatment had a significant effect on trapping of SSRBCs in the lungs, liver and spleen compared to sham-treated cells. *: *p*<0.001 compared to sham-treated SSRBCs. Error bars show SEM of three different experiments.

I examined the effect of MEK inactivation on human SSRBC trapping in organs typically affected and damaged in SCD, including the lungs, liver, spleen and kidneys. Trapping of sham-treated SSRBCs was extensive in the lungs, liver and spleen (Figures 5A and 5B). However, treatment of SSRBCs with RDEA119 significantly decreased human SSRBC sequestration in the lungs, liver, and spleen. SSRBC trapping in the kidneys was similar in animals infused with sham-treated and RDEA119-treated SSRBCs (Figures 5A and 5B). This suggests that MEK-dependent ERK1/2 inhibition in SSRBCs reduces SSRBC organ trapping, which may in fact result in reduced organ damage in SCD.

### MEK inhibition in SSRBCs increases the number of human SSRBCs in bloodstream

**Figure 6:**
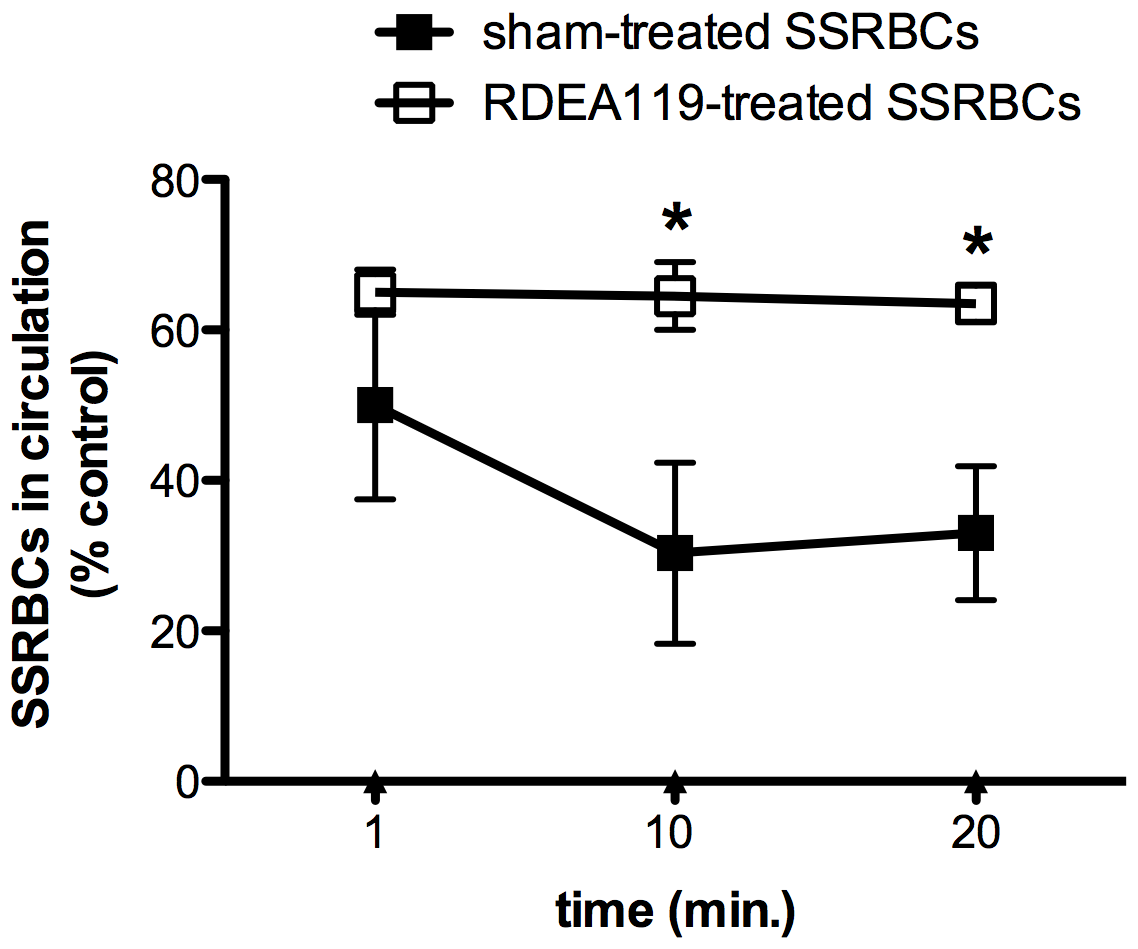
RDEA119 enhances the percentage of SSRBCs circulating in bloodstream. Anesthetized mice were injected with fluorescence-labeled sham- (▪) or fluorescence-labeled RDEA119-treated SSRBCs (□) (n = 3 for each treatment). Blood samples were collected from sham-treated and RDEA119-treated SSRBCs after 1, 10 and 20 min of human SSRBC infusion. Error bars show SEM of three different experiments. A significantly greater percentage of RDEA119-treated than sham-treated SSRBCs was retained in the circulation at 10 and 20 min post SSRBC infusion. *: *p*<0.05 compared to sham-treated SSRBCs.

The effect of MEK inhibition on human SSRBCs in circulation in vessels and arterioles of animals was also examined. The percentage of sham-treated human SSRBCs that did not adhere to the vascular walls and remained circulating in bloodstream of animals decreased over time (Figure 6). However, RDEA119-treated human SSRBCs circulating in vessels and arterioles of animals, was higher than sham-treated SSRBCs, and did not decrease over time. The percentage of RDEA119-treated SSRBCs in bloodstream 10 min post-infusion of SSRBCs was 2.1-fold higher than sham-treated SSRBCs [*p*<0.05 for RDEA119-treated vs. sham-treated at 10 min or 20 min]. These data indicate that inhibition of MEK-dependent ERK1/2 signaling is associated with an increase in the number of SSRBCs circulating in vessels and arterioles of animals compared to sham-treated cells, and strongly argue that such increase in the number of MEK inhibitor-treated SSRBCs circulating in bloodstream reflects the fact that these sickle cells did not adhere to the vascular endothelium and were not trapped in organs.

## Discussion

Acute painful vasoocclusive crises caused largely by sickle red cell adherence [38]–[40] in patients with SCD, remain a major scientific challenge that invites conceptually new approaches. I now provide a potential new therapeutic alternative to reduce acute vasoocclusive crises. Recently, we have discovered that although MEK and ERK1/2 are highly expressed in both SSRBCs and normal RBCs, ERK1/2 is abnormally activated only in SSRBCs, but not in normal RBCs [18]. To determine the therapeutic potential of SSRBC MEK-dependent ERK1/2 signaling inactivation, I have selectively targeted MEK using MEK inhibitors that have been already developed for human use. My present data reveal a potential contribution of SSRBC MEK-dependent ERK1/2 activation in the vasoocclusive process, by which human SSRBCs both adhere to the vascular endothelium and act as a stimulus activating adhesion of human PMNs to normal endothelial cells.

I show that SSRBC MEK-dependent ERK1/2 inactivation with MEK inhibitors significantly decreased SSRBC adhesion to both non-activated and activated microvascular ECs *in vitro*. My *in vivo* data further provide functional evidence that SSRBC MEK-dependent ERK1/2 inactivation prevents both SSRBC adhesion and vasoocclusion. Whereas mice infused with sham-treated human SSRBCs experienced rapid and significant SSRBC adhesion in enflamed venules and arterioles promoting dramatic vasoocclusion, treatment of SSRBCs with the MEK inhibitors, U0126 and RDEA119, effectively reduced SSRBC adherence to activated ECs and vasoocclusion. MEK-dependent ERK1/2 signaling inactivation clearly not only reduced SSRBC adhesion and vascular blockade but also decreased SSRBC trapping in organs typically affected in SCD, including the spleen, liver and lungs. In contrast, normal RBCs sham-treated or MEK inhibitor-treated showed no real adherence to enflamed vessels. This suggests that differential regulation of one or more steps in the MEK-dependent ERK1/2 signaling cascade in SSRBCs vs. normal RBCs may account for adherence of SSRBCs, but not normal RBCs.

I observed SSRBC adhesion occurring both in enflamed vessels, predominantly in small and large postcapillary venules promoting vasoocclusion, and in arterioles [41]. Because only SSRBCs, but not normal RBCs, adhered to both activated human and murine microvascular endothelial cells *in vitro* (Figure 1) and *in vivo* (Figure 3), interactions between human SSRBCs and murine endothelium *in vivo* were not due to non-specific binding or TNFα effect on SSRBC adherence, since treatment of SSRBCs with TNFα failed to increase SSRBC adhesion to endothelial cells *in vitro* (data not shown). I also recognize that the characteristics of endothelial cells and microcirculatory beds vary among different tissues; it remains to be determined whether SSRBC adhesion is equally avid in all tissues. In addition, my model represents an appropriate system for identifying the effect of signaling mechanisms in SSRBCs involved in adhesion and vasoocclusion *in vivo*, since it allows for the manipulation of RBCs exclusively, while still allowing study of adhesion and vasoocclusion in an intact vasculature, in the context of physiologic blood flow and shear stresses, and where vessels were non-instrumented and contralateral to the window chamber.

Numerous mechanisms of SSRBC adhesion to endothelium and vasoocclusion have been demonstrated in *in vitro* and *ex vivo* models, but there are only limited data regarding the contribution of specific factors initiating or inducing those interactions *in vivo*. Studies have previously shown that human SSRBCs adhere to human, murine and rodent endothelial αvβ3 [14], [17], [42], [43]. There is also a growing body of data showing that activated monocytes [33] and PMNs [44] adhere to endothelial cells in SCD. Turhan et al. for instance have demonstrated in transgenic sickle mice that murine SSRBCs can bind to adherent leukocytes in inflamed cremasteric vessels, producing vasoocclusion [30]. In my animal model, administration of TNFα to nude mice prior to SSRBC infusion activates murine neutrophil adherence to the endothelium. It is therefore possible that SSRBCs adhered to both activated endothelial cells and leukocytes promoting vasoocclusion, and MEK inhibitors may affect SSRBC-vascular endothelium and SSRBC-leukocyte interactions, and subsequent vasoocclusion. I further surmise that MEK inhibitors may alleviate vascular and organ injuries at the site of SSRBC-induced vasoocclusion, since these inhibitors reduced both SSRBC adhesion and SSRBC organ trapping; an effect associated with enhanced SSRBC fraction circulating in bloodstream.

Furthermore, MEK inhibitors have the potential to prevent SSRBCs from activating neutrophils to adhere to endothelium. While SSRBCs stimulated adhesion of naïve neutrophils to non-activated endothelial cells, normal RBCs failed to activate naïve PMN adherence. Increased PMN adhesion is unlikely to be caused by SSRBC-induced endothelial changes, which can occur in response to contact with SSRBCs [45], because contact of SSRBCs with non-activated or TNFα-activated ECs failed to cause increased adhesion of naïve PMNs to ECs (Figure 2D). In addition, hypotonic lysis of SSRBCs prior to PMN adhesion assays did not lower adhesion of PMNs to non-activated ECs (data not shown). These data are in concordance with our previous data showing that human SSRBCs are able to increase adhesion of monocytes and lymphocytes to ECs *in vitro* [16], and murine leukocytes to the vascular endothelium in nude mice *in vivo* [17]. My data now further show that SSRBCs’ ability to activate PMN adhesion to ECs was completely inhibited by 4 different MEK inhibitors. I therefore suggest that abnormal MEK-dependent ERK1/2 activation in SSRBCs could contribute to the pathophysiology of SCD by activating a secondary cellular process: neutrophil adherence to the endothelium. Previous studies have also shown that activated neutrophils can increase SSRBC adhesion to lung vascular endothelium [46], [47]. It is therefore possible that activated neutrophils may affect SSRBC adhesiveness by enhancing SSRBC ERK1/2 activation, a mechanism yet to be defined.

Activation and adherence of PMNs could induce production of multiple cytokines, including TNFα, IL-8 and IL-1β, that activate the vascular endothelium, resulting in up-regulation of cellular ligands, including intracellular adhesion molecule-1 (ICAM-1) and vascular cell adhesion molecule-1 (VCAM-1), for blood cell adhesion molecules. Such processes could further enhance the adhesiveness of inflammatory cells, platelets and SSRBCs to endothelium [48], as well as further tissue damage. These events collectively could be followed by activation of both coagulation cascade as evidenced previously by elevated plasma levels of thrombin-antithrombin (TAT) complexes, prothrombin fragment 1.2 (F1.2), and D-dimer [49], [50], as well as increased platelet activation [51] in patients with SCD, all of which contribute to a systemic hypercoagulable state [52], and inflammatory pathways.

In summary, anomalous MEK-dependent ERK1/2 activation in SSRBCs can contribute to the pathogenesis of SCD by potentially activating at least two significant cellular players of vasoocclusive events, SSRBC adhesion and SSRBC-induced activation of leukocytes. Thus, MEK inhibitors could have a novel multi-facetted therapeutic application for acute vasoocclusive episodes, and my data are a formidable initial step towards testing of existing MEK inhibitors, such as RDEA119 and the FDA-approved trametinib in SCD.

## Supporting Information

Movie S1 **Adhesion of sham-treated human SSRBCs** ***in vivo***. Movies were merged to show SSRBC circulatory behavior in the vasculature at different locations and over time. Sham-treated SSRBCs showed extensive adhesion in post-capillary vessels and arterioles, and progression of vasoocclusion occurred within the first 2 to 3 minutes following human SSRBC infusion, which resulted in permanent blockade of some venules both at junctions and straight segments causing blood flow stasis.
(MOV)

Movie S2 **Adhesion of RDEA119-treated human SSRBCs** ***in vivo*** **.** Movies were merged to show SSRBC circulatory behavior in the microvasculature at different locations and over time. Treatment of SSRBCs with RDEA119 reduced both SSRBC adhesion to the vessel and arteriole walls and vasoocclusion.
(MOV)

Movie S3 **Adhesion of sham-treated human normal RBCs** ***in vivo*** **.** Sham-treated normal RBCs failed to adhere to enflamed vessels and promote vasoocclusion.
(MOV)
